# Inhibitory effects of *Serjania erecta* on the development of *Chrysodeixis includens*

**DOI:** 10.1038/s41598-022-19126-3

**Published:** 2022-08-29

**Authors:** Samylla Tassia Ferreira de Freitas, Agna Rita dos Santos Rodrigues, Ana Cláudia Cardoso Ataídes, Gisele Cristina de Oliveira Menino, Giselle Santos de Faria, Luciana Cristina Vitorino, Fabiano Guimarães Silva, Fábio Henrique Dyszy

**Affiliations:** 1 Programa de Pós-Graduação em Ciências Agrárias, Instituto Federal de Educação, Ciência e Tecnologia Goiano (IF Goiano, Campus Rio Verde), Rodovia Sul Goiana, Km 01, Zona Rural, Rio Verde, GO 75901-970 Brazil; 2 Instituto Federal de Sergipe (Campus Itabaiana), Avenida Padre Airton Gonçalves Lima, 1140 São Cristóvão, Itabaiana, 49500-543 Brazil; 3 Programa de Pós-Graduação em Agroquímica, Instituto Federal de Educação, Ciência e Tecnologia Goiano (IF Goiano, Campus Rio Verde), Rodovia Sul Goiana, Km 01, Zona Rural, Rio Verde, GO 75901-970 Brazil; 4 Programa de Pós-Graduação em Biodiversidade e Conservação, Instituto Federal de Educação, Ciência e Tecnologia Goiano (IF Goiano, Campus Rio Verde), Rodovia Sul Goiana, Km 01, Zona Rural, Rio Verde, GO 75901-970 Brazil; 5 Instituto Federal Goiano – Campus Rio Verde, Rodovia Sul Goiana, Zona Rural, s/n, Rio Verde, GO 75901-970 Brasil

**Keywords:** Natural product synthesis, Toxicology

## Abstract

The soybean looper, *Chrysodeixis includens*, is a primary soybean pest that reduces crop productivity. This work examined control of *C. includens* populations with methanolic extract of *Serjania erecta*, a native Cerrado plant, while minimizing risks to pollinators, natural enemies and the environment. *Serjania erecta* specimens were collected, identified, and subjected to methanol extraction. Bioassays were performed using newly hatched and second-instar caterpillars and different extract concentrations on the diet surface to obtain IC_50_ values. Two replicates, containing 10 caterpillars, were established in triplicate. The IC_50_ values were 4.15 and 6.24 mg of extract mL^−1^ for first-instar and second-instar caterpillars, respectively. These growth inhibition results informed the extract concentrations assessed in subsequent development inhibition assays, in which the pupal weight was higher under the control than under the treatments. Extract treatments increased the duration of the larval, pupal and total development. The potential of different concentrations of *S. erecta* extract to inhibit the enzymes carboxylesterases was also evaluated. Carboxylesterases activity decreased by 41.96 and 43.43% at 7.8 and 15.6 μg mL^−1^ extract, respectively. At 31.3 μg mL^−1^ extract, enzymatic activity was not detected. Overall, *S. erecta* leaf methanolic extract showed inhibitory potential against carboxylesterases.

## Introduction

The soybean looper moth, *Chrysodeixis includens* (Walker) (Lepidoptera: Noctuidae), is a caterpillar with a defoliating habit^1,2^ and is considered a primary pest of soybean crops. The biological control of *C. includens* was reduced following the application of fungicides for the control of the fungus (*Phakopsora pachyrhizi*) responsible for Asian soybean rust^2^. While the control of *C. includens*is based on the application of synthetic insecticides, these products are known to have deleterious effects also on nontarget organisms^3,4^.

Botanical insecticides are produced from plant extracts or essential oils. They are composed of mixtures of secondary metabolites and are produced using solvents of different polarities and different plant parts^5,6^. These insecticides are degraded relatively easily and are much less likely to select for resistance in the target species^7^.

Botanical insecticides have been shown to inhibit enzymes, including alpha-amylase, alpha-glucosidase of *Spodoptera litura*^8^, acetylcholinesterase, carboxylesterases, and glutathione-S-transferase of *Brontispa longissima*^9^. Blocking the activity of these enzymes can slow down the digestive process and damage the functioning of the insects' nervous system, causing them to die^8,10,11^. The susceptibility of insects to these agrochemicals is also related to the inhibition of detoxification pathways, normally stimulated by the action of acetylcholinesterase, glutathione-S-transferase and carboxylesterases^12,13^. Ali et al.^14^ demonstrated that the inhibition of carboxylesterases, induced by plant extracts in insects, caused high mortality and impacted the lethality of the offspring. The detoxifying effect of carboxylesterases is related to the ability to hydrolyze acids and alcohols and degrade harmful substances^15^. It is known that high expression or genetic mutations involving the production of these enzymes affect insect resistance to insecticides^16^. In addition to enzyme inhibition, other effects of botanical insecticides on insects include morphological deformities on *S. litura*^8^, antinutrient action on *B. longissima*^9^, mortality of *Helicoverpa armigera*, *S. litura*, *B. longissima* and *Sitophilus zeamais*^6,8,9,17^, repellence against *S. zeamais* and *H. armigera*^17,18^, inhibition of growth on *H. armigera*^6^, ovicide against *Rhodnius prolixus*^19^, and delayed development of *S. litura* and *B. longissima*^8,9^.

At least 12,000 higher plants are estimated to occur in the Brazilian Cerrado savanna^20^. *Serjania erecta*, known as the five-leaf vine, is native to the Cerrado and produces numerous secondary metabolites, such as saponins, flavonoids, tannins, and terpenes^21^, which are components contributing to the high insecticidal activity observed in plants of the Sapindaceae^22^.

One of the most effective ways to minimize the loss of biodiversity is the development of conservation programs and the sustainable use of biological resources^23^. Given the biotechnological potential of *S. erecta*, it is important to guarantee its conservation. This plant may provide compounds that control *C. includens*, contributing to a reduction in the application of synthetic insecticides. The present study investigated the weight of newly hatched *C. includens* caterpillars and second-instars, the development of the immature stages following exposure to the methanolic extract of *S. erecta* leaves, and the effects of this extract on carboxylesterases enzymes.

## Materials and methods

### Insect source

The initial population of *C. includens* was established from pupae reared in the laboratory and provided by the ESALQ-USP Entomology Laboratory. As this study did not use caterpillars collected from natural populations, however, all necessary licenses and permissions were obtained for the development of the study, including a license to collect (SISBio - 769521) and permission for in vitro cultivation (ordinance 265 of 07/22/19 of the IFGoiano campus Rio Verde). The caterpillars were raised in the Entomology Laboratory of the Rio Verde campus of the Goiano Federal Institute at 25 ± 2 °C, a relative humidity of 60 ± 10%, and a 14-h photophase.

### Insect rearing

The caterpillars were raised on an artificial diet based on white bean^24^. The adults were housed in enclosures made from PVC tubes (20 cm in length and 15 cm in diameter) on a 20-cm-diameter plastic base plate and covered with organza-type fabric held in place with an elastic band. The enclosures were lined with sheets of A4 paper as a substrate for egg laying. A 20 mL container with a wad of cotton soaked in a 10% honey solution was added to each enclosure. The enclosures were cleaned and provisioned daily when any eggs were removed. Once hatched, the caterpillars were maintained in pairs in 50 mL plastic containers, with a portion of the feed (1.5 cm × 1.5 cm). These containers and the feed were changed twice a week. The pupae were removed and housed in 500 mL plastic containers until emergence of the adults, which were transferred to the enclosures described above. The *C. includens* populations were maintained in isolation at a temperature of 25 ± 2 °C and relative humidity of 60 ± 10% with 14-hourphotophase.

### Collection of plant material

The plant material (healthy leaves of *S. erecta*) was collected from the Fontes do Saber farm in Rio Verde, Goiás, Brazil (− 17.783262° S − 50.967928° O), and a voucher specimen was deposited in the herbarium of the Rio Verde in Goiano Federal Institute under catalog number 545. The material was then sent to Dr. Germano Guarim Neto at the Federal University of Mato Grosso for identification. The identification of the material as *Serjania erecta* Radlk was confirmed. The handling and use of plants and caterpillars followed institutional guidelines.

### Acquisition of the extract

The leaves of *S. erecta* were dried for 5 days at 40 °C in an air-circulated Marconi MA 035 stove until reaching a constant weight. The samples were then triturated to a powder in a Willey Tecnal TE 680 knife mill. The extracts were obtained from this powder, following the methodology proposed by Fernandes et al.^21^. Thus, 1 L of methyl alcohol was added per 200 g of powder. This mixture was maintained in sealed Erlenmeyer flasks for 24 h and then filtered. The filtered samples were processed in a Tecnal TE-210 rotary evaporator at 65 °C, a temperature close to the boiling point of the solvent. The solvent recovered at the end of the process was added to the pellet resulting from the initial extraction, and the whole procedure was repeated twice. The extract was then transferred to a lidded glass container to be frozen and lyophilized in a Liotop L108. The lyophilized product was stored at ambient temperature until the bioassays.

### Assays of the inhibition of caterpillar growth

Newly hatched and second instar *C. includens* caterpillars were exposed to five different concentrations of the extract: 1.25 mg L^−1^, 2.5 mg L^−1^, 5.0 mg L^−1^, 10.0 mg L^−1^, and 20.0 mg L^−1^ (v/v). The control treatment was distilled water with 2.5% methyl alcohol (v/v). All bioassays were run twice in triplicate. For each concentration, 300 μL of the extract solution was applied to the surface of 10 mL of the artificial diet, and the diet was placed in 100 mL plastic containers. The solution was then spread over the surface of the feed with circular movements. These containers were left without lids for two hours until all the excess moisture had dried off. Subsequently, 10 newly hatched caterpillars or second instars were transferred to each container, which was then closed and labeled with the age of the insect and the concentration of the extract. After 7 days, the surviving caterpillars were weighed on an analytical balance, and the mean weight of the caterpillars was obtained for each concentration of the extract. The mortality of the caterpillars was also quantified.

### Assays of the inhibition of caterpillar development

The results of the caterpillar growth inhibition assays were used to define the concentrations of *S. erecta* leaf extract to be evaluated in the caterpillar development inhibition assays, i.e., 10 mg L^−1^ and 20 mg L^−1^ of extract. The assays were based on similar procedures, with the methanolic extract of *S. erecta* being diluted in distilled water containing 2.5% (v/v) methyl alcohol. In this case, however, five repetitions were conducted per concentration, in addition to the control, which was distilled water containing 2.5% (v/v) methyl alcohol. After 7 days, the caterpillars were removed from the containers with the extract applied to the surface of the feed. They were then placed in individual containers and fed continuously on the artificial diet with no added extract. The duration of the larval and pupal stages and the timing of the emergence of the adults were determined through daily monitoring of the containers. The pupae were sexed to verify the sex ratio.

### Extraction of carboxylesterases

Initially, five samples, each containing five fourth-instar caterpillars of *C. includens,* were obtained from the Agricultural Entomology Laboratory of IF Goiano, campus Rio Verde. For the extraction of carboxylesterases, the caterpillars were dissected in saline solution, and later, their abdomens were homogenized in 50 μL of sodium phosphate buffer (0.02 M, pH 7.0). After homogenization, 950 μL of sodium phosphate buffer (0.02 M, pH 7) was added, and the homogenate was centrifuged at 10.000 RCF for 15 min at 4 °C. The supernatant was collected and frozen for further protein quantification. Total proteins were determined using the bicinchoninic acid method with bovine serum albumin (BSA) as the standard^25^.

### Enzyme activity assays

The activity of carboxylesterases (EC 3.1.1.1) was evaluated by adapting the van Asperen method, with *p*-nitrophenyl-acetate solubilized in DMSO as substrate, using 96-well microtiter plates. Carboxylesterases degrades *p*-nitrophenyl acetate to acetate and *p*-nitrophenol. The increase in absorbance of the *p*-nitrophenol product was monitored in a plate reader (Versamax, Molecular Devices), with λ = 405 nm, for 10 min at each substrate concentration. The readings, taken in triplicate, from each well were monitored at intervals of 8 s, totaling 76 readings per well. Steady-state enzyme kinetics assays were performed following the Michaelis–Menten model, keeping the substrate consumption rate within a maximum of 10% of the final substrate concentration at each concentration studied. The substrate concentrations ranged from 7.8 to 500.0 µM, and the assays were conducted in 0.02 M sodium phosphate buffer, pH 7.0, at 20 °C ± 2 °C, with a concentration of total proteins of 1 g.

### Enzyme inhibition assays

For the inhibition assays, dilutions of the methanolic extract of *S. erecta* were prepared in 0.02 M sodium phosphate buffer, pH 7.0, at final concentrations ranging from 0.0078 mg mL^−1^ to 2.0 mg mL^−1^ inhibitor. In this buffer containing the inhibitor, enzymes were diluted to a final concentration of 1 µg of total protein. Substrate concentrations and other data acquisition conditions were identical to those described in the “Enzyme activity assays”.

### Statistical analyses

The data on the weight of the caterpillars exposed to the different concentrations of methanolic *S. erecta* leaf extract were adjusted to a logistic nonlinear regression model with three parameters based on the formula y = {a/[1 + (x + IC_50_)^b^]}, where *y* = the mean weight of the caterpillars in mg, *x* = the concentration of the extract, *a* = the difference between the maximum and minimum points of the curve, *b* = the curve slope, and IC_50_ = the concentration that results in a 50% reduction in the weight of the insect.

The data on the weight of the pupae, the duration of the larval and pupal phases, total development, and viability of the immature moths were first evaluated for normality using the Kolmogorov test (SAS Proc Univariate) and for homogeneity using Bartlett’s test and subsequently submitted to analysis of variance. Significant differences between the means were compared by the Tukey test at 5% probability. Similar tests were applied to the enzyme activity data.

### Ethics Statement

This study did not use any vertebrate animal.

## Results and discussion

To test the hypothesis that the methanolic extract of *S. erecta* leaves influences the development of *C. includens* caterpillars, these animals were fed diets with or without the extract for 7 days. Caterpillars of different ages were then weighed to determine the potential inhibition of growth. Development inhibition trials were then run using recently hatched caterpillars, which were presented with diets with/without the extract for 7 days, after which they were given a normal diet without the extract. During these trials, the mean duration of the larval and pupal stages and the full developmental period were recorded, and the viability of the immature stages was determined.

The body mass data were adjusted to a logistic nonlinear regression model with three parameters to estimate the inhibition of growth, IC_50_, as shown in Table 1. The IC_50_ value was significantly higher for the second-instars (t = 1.0021_14,25_; *P* = 0.0444) than for the newly hatched caterpillars. Based on the ratio of the IC_50_ values recorded in the two groups, the extract was 1.5 times more potent to newly hatched caterpillars than to the second-instars.

**Table 1:** Parameters of the nonlinear logistic regression plotted to estimate the patterns of weight reduction in newly hatched and second-instar *Chrysodeixis includens* caterpillars exposed to different concentrations of methanolic extract of *Serjania erecta* leaves.

Caterpillar age	a^1^	b^2^	IC_50_ ± SEM^3^	Power	R^2^	*p*
Neonate	0.01 ± 0.0	3.89 ± 0.94	4.15 ± 0.32	1.51	0.9918	0.04002
Second-instar	0.05 ± 0.001	1.96 ± 0.13	6.24 ± 0.26	–	0.9988	0.00432

^1^*a* corresponds to the difference between the minimum and maximum points of the curve;

^2^*b* corresponds to the curve slope;

^3^SEM corresponds to the standard error of the mean.

**Figure 1:**
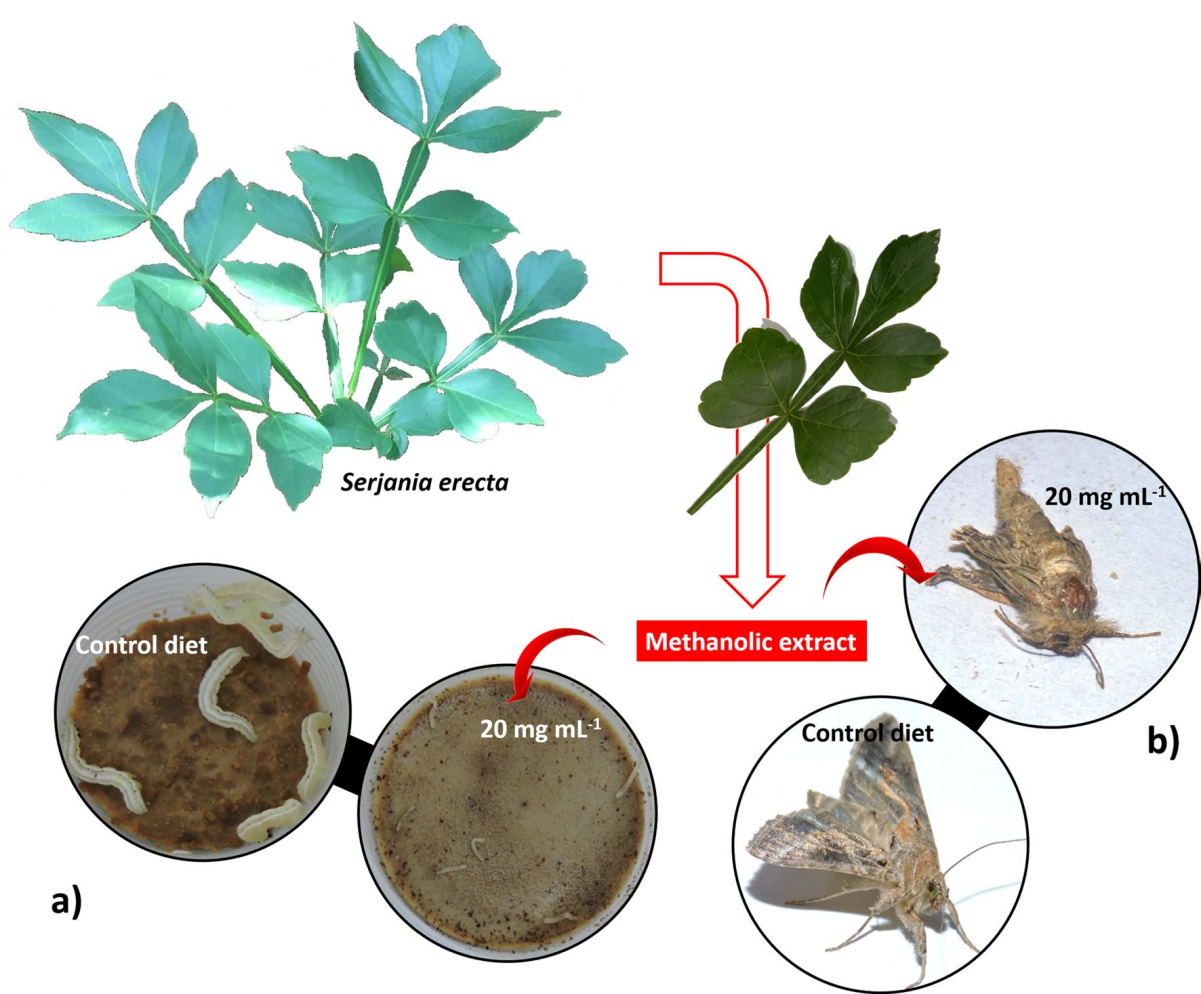
Caterpillars of *Chrysodeixis includens* exposed to the control diet and a diet containing the methanolic extract of *Serjania erecta* leaves at a concentration of 20 mg mL^−1^ (**a**) and normal *Chrysodeixis includens* adults and morphologically deformed individuals (right) exposed to methanolic extract (**b**).

The maximum growth inhibition was 92% in the second-instar caterpillars exposed to an extract concentration of 20 mg mL^−1^ (Fig. 1a). In the development inhibition trials, significant differences were found between treatments in the duration of the larval (F_2,12_ = 155; *p* < 0.001) and pupal (F_2,12_ = 28.84; *p* < 0.001) stages, total developmental time (F_2,12_ = 242.36; *p* < 0.001), and pupal weight(F_2,12_ = 9.57; *p* < 0.0033). However, no significant variation was recorded in the viability of the immature moths (F_2,12_ = 0.49; *p* = 0.6258). In addition to these other patterns, morphological alterations (underdeveloped wings) were also observed in individuals exposed to an extract concentration of 20 mg mL^−1^ (Fig. 1b).

The methanolic leaf extract of *S. erecta* was 1.5 times more potent to newly hatched *C. includens* caterpillars than to older individuals, although biologically, this difference may not have any major implications. These findings are consistent with existing data, which indicate higher mortality in first-instar *C. includens* caterpillars following exposure to the unrefined extract of *Annona mucosa* seeds than in third- and fifth-instar caterpillars^26^. Other studies have attributed the greater susceptibility of younger caterpillars to the reduced effectiveness of detoxifying intestinal enzymes in these herbivorous lepidopterans^27^. In the present study, the 10 mg mL^−1^ and 20 mg mL^−1^ concentrations of the extract reduced the mass of newly hatched caterpillars by 92% and 93%, respectively. However, no significant difference was recorded in the mortality of the control and treatment groups (Table 2).

**Table 2:** Influence of the different concentrations of the methanolic extract of *Serjania erecta* leaves on the development of *Chrysodeixis includens*.

Treatment	Duration of the larval phase	Duration of the pupal phase	Total developmental duration	Weight of the pupa	Viability of the immature insect
Control	16.9 ± 0.06b^1,2^	8.07 ± 0.10b	25 ± 0.06b	0.24 ± 0.004a	60.32 ± 4.4a
10 mg mL^−1^	23.5 ± 0.36a	8.94 ± 0.06a	32.4 ± 0.34a	0.22 ± 0.002b	49.40 ± 9.96a
20 mg mL^−1^	24.1 ± 0.42a	8.99 ± 0.12a	33.1 ± 0.36a	0.22 ± 0.005b	48.5 ± 11.7a

^1^Different letters in the same column indicate a significant difference (*p* < 0.05) between the values based on Tukey test;

^2^values after " ± " are the standard error of the mean.

The 10 mg mL^−1^ and 20 mg mL^−1^ extract concentrations delayed larval, pupal and total development significantly in comparison with the control (Table 2). The body mass also decreased significantly in both treatments in comparison with the control (Table 2), although viability was not significantly different in any of the analyses. The development of the caterpillars returned to normal once the individuals were transferred to a normal diet (without the extract), although their delayed developm ent and reduced weight may make them more susceptible to other factors, such as predators and sunlight. The sublethal effect of underdeveloped wings may be associated with a reduction in longevity, as well as reduced reproductive success, due to its influence on other aspects of the life of the insect, such as alterations of its immune system and behavior^4^.

The delayed development and weight loss observed in *C. includens* may have been related to the phenolic compounds found in the *S. erecta* extract, including kaempferol^28^, saponins^29^, and apigenins^28^. Phenolic compounds may cause a significant reduction in the survival and growth of insects due to the inactivation of digestive enzymes^30^. Caterpillars of *Pieris brassicae* (Lepidoptera) exposed to a diet treated with kaempferol suffered a significant reduction in body mass^31^. Extracts of *Sapindus mukorossi* (Sapindaceae), which include saponins, caused a reduction in the feeding of *Thysanoplusia orichalcea* (Lepidoptera) and inhibited both larval and pupal development^32^, which may have been related to the hydrolysis of the saponins into aglycons, which are liberated by the glycosylases found in the intestine of the insect^32,33^. Cipollini et al.^34^ showed that purified apigenin reduced feeding in *Spodoptera exigua* (LepidopteraNoctuidae) caterpillars and may be capable of interrupting the molting cycle^35^.

**Figure 2:**
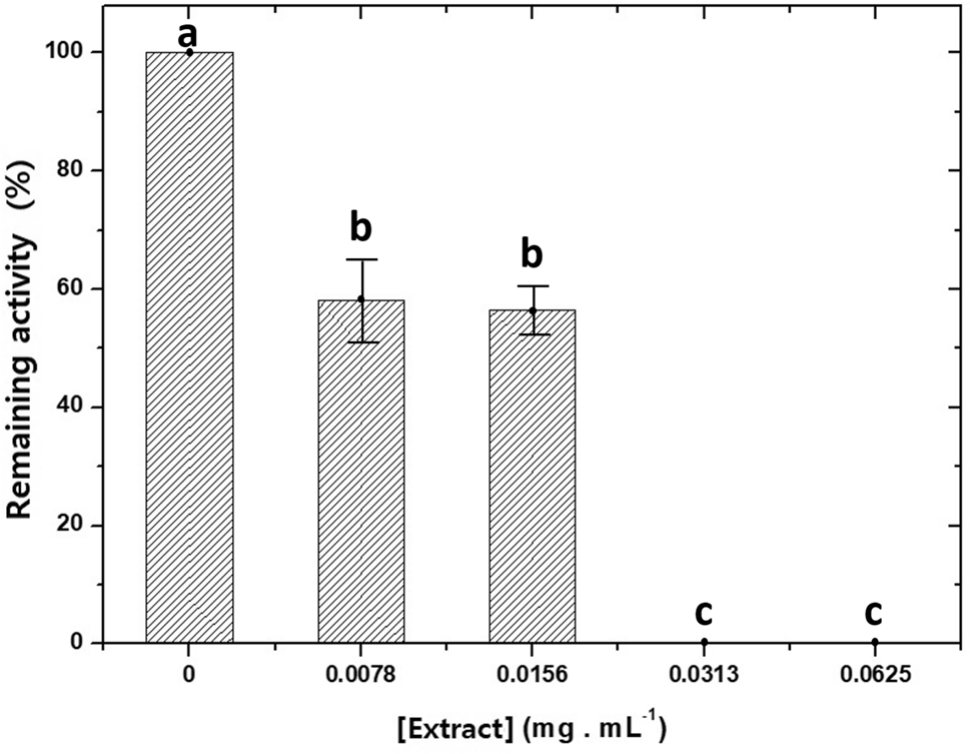
Remaining activity of carboxylesterases extracted from the intestines offourth-instar larvae of *Chrysodeixis includens* in the presence of 500 µM of substrate at different concentrations of the methanolic extract of *Serjania erecta* leaves. Different letters indicate a significant difference (*p* < 0.05) between the values based on Tukey test.

When verifying the enzymatic activity in the presence of 500 µM of substrate, at concentrations of 7.8 and 15.6 µg mL^−1^ of extract, the activity of carboxyl-esterases decreased by 41.96 and 43.43%, respectively (Fig. 2). At a concentration of 31.3 µg mL^−1^ of *S. erecta* leaf methanolic extract, it was not possible to detect enzymatic activity.

The inhibitory effect of plant extracts has been known for a long time^36^. In these works, secondary metabolites such as saponins, flavonoids, tannins and terpenes seem to act in different ways on the activity of enzymes important for the metabolism of various organisms^8,37,38^.

Regarding *S. erecta*, there are few reports in the literature regarding the inhibitory activity of its extracts. Broggini et al.^29^ showed the inhibitory effect of crude extracts of this species using thin layer chromatography. In this work, the flavonoid fraction of this extract was shown to be more selective for inhibition of acetylcholinesterase than for that of butyrylcholinesterase, while the saponin fraction showed greater inhibition of butyrylcholinesterase than of acetylcholinesterase^29^. Thus, the presence of metabolites in the extract of this plant, at a concentration of 31.3 µg mL^−1^, is high enough to totally inhibit the activity of carboxyl-esterases.

**Figure 3:**
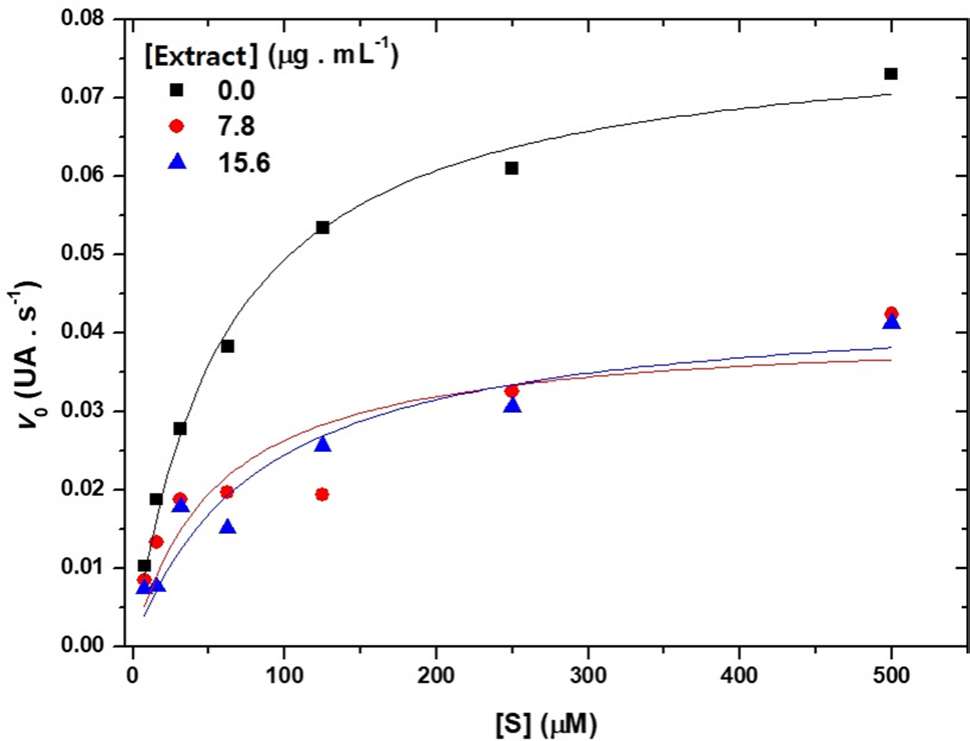
Kinetic profile of carboxylesterases extracted from the intestines of fourth-instar *Chrysodeixis includens* larvae in the absence and presence of 7.8 and 15.6 µg mL^−1^ *Serjania erecta* leaf methanolic extract (20 mM sodium phosphate buffer, pH 7.0, T = 22 °C).

In the present work, to characterize the kinetic behavior of these enzymes in the absence and presence of the methanolic extract of *S. erecta* leaves, steady-state kinetics experiments were carried out, following the Michaelis–Menten model (Fig. 3).

Analyzing the results according to the Michaelis–Menten model, it is possible to obtain the maximum velocity of the enzyme (Vmax), as well as its affinity constant (Michaelis constant, KM), as shown in Table 3.

**Table 3:** Vmax (UA/s) and KM (μM) of carboxylesterases extracted from intestines of fourth-instar larvae of *Chrysodeixis includens* in the absence and presence of 7.8 and 15.6 μg mL^−1^ of methanolic extract (20 mM sodium phosphate buffer, pH 7.0, T = 22 °C) from *Serjania erecta* leaves.

[Extract] (μg mL^−1^)	R^2^	*V*_max_ (UA/s)	*K*_M_ (μM)
0.0	0.99027	0.07890 ± 0.00265^1^	59.59000 ± 6.35617
7.8	0.75802	0.04057 ± 0.00631	53.80821 ± 27.31495
15.6	0.89753	0.04426 ± 0.00549	80.50957 ± 29.27428

^1^Values after “ ± ” are the standard error of the mean.

**Figure 4:**
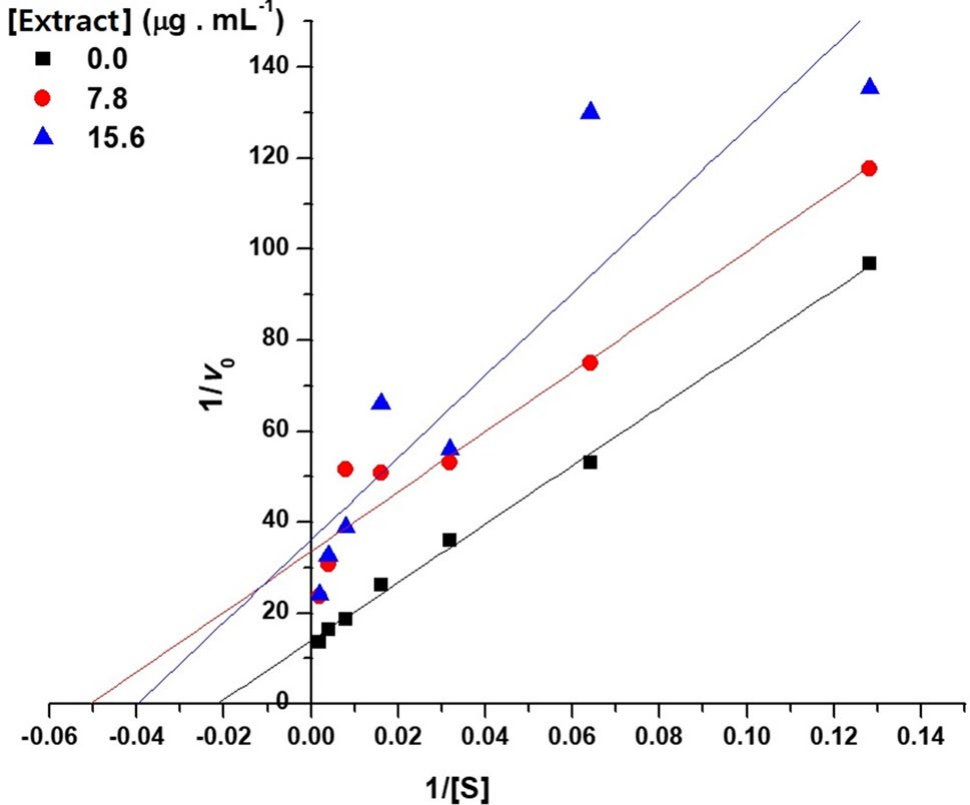
Graph of the double reciprocals for carboxylesterases extracted from the intestines offourth-instar larvae of *Chrysodeixis includens* in the absence and presence of 7.8 and 15.6 µg mL^−1^ of methanolic extract of *Serjania erecta* leaves (20 mM sodium phosphate buffer, pH 7.0, T = 22 °C).

In the presence of the extracts, the enzymatic activity is disturbed to the point of not allowing a good adjustment of the experimental data to the theoretical curve, reflected in high errors, mainly with regard to the determination of KM and R^2^. However, it can be noted that in the presence of the extracts, a decrease in Vmax and, at least at the lower concentration of extract, a maintenance of the KM values could indicate a mixed-type inhibition. This can be seen in the graph of reciprocal doubles, shown in Fig. 4, which shows typical mixed inhibition behavior.

Overall, the results of the present study indicate that the insecticidal effects of the methanolic extract of *S. erecta* leaves on *C. includens* are probably related to the inhibition of the enzymes of the digestive tract, which reduces the absorption of nutrients, thereby impacting the development of the caterpillars. However, we cannot entirely rule out the possible inhibitory action of the extract on other target enzymes, including those of the central nervous system such as acetylcholinesterase.

## Data Availability

Data may be made available by contacting the corresponding author.
